# The Long Shadow of Job Loss: Britain's Older Industrial Towns in the 21st Century

**DOI:** 10.3389/fsoc.2020.00054

**Published:** 2020-08-19

**Authors:** Christina Beatty, Steve Fothergill

**Affiliations:** Centre for Regional Economic and Social Research, Sheffield Hallam University, Sheffield, United Kingdom

**Keywords:** industry, towns, employment, welfare benefits, UK

## Abstract

This article takes a long view of economic change in Britain's older industrial towns, drawing on the authors' accumulated research into labor market trends in the places and communities most affected by deindustrialization. It begins by documenting the industrial job losses over the last 50 years and their impact on unemployment, economic inactivity and welfare benefit claims, highlighting the diversion onto incapacity benefits triggered by job loss that remains a major feature of the towns. It then looks at the evidence on the present-day labor market in the towns, identifying job growth at a slower pace than in the cities and continuing weaknesses in terms of earnings, qualifications and occupational mix. These are the on-going problems the authors describe as the ‘long shadow of job loss’. The evidence also shows that despite years of job loss, industry remains a key component of the towns' economy and that the towns are increasingly connected to surrounding areas, including nearby cities, by strong commuting flows.

## Aim of the Paper

A widely-held view of the UK economy is that it has become ‘deindustrialized.’ The world's first industrial nation now employs far fewer workers in manufacturing and mining than was the case fifty or more years ago and the economy as a whole is now dominated by jobs and output in the service sector. That this deindustrialization has happened in the UK is indisputable but the massive consequences for the individuals and communities that once depended upon industry for their livelihood are only poorly understood.

This is the gap in knowledge that the present paper helps to fill by bringing to bear statistical evidence on the contemporary labor market in the places hardest hit by deindustrialization. In doing so, the paper is also intended to provide a context for the others in this volume.

Specifically, the paper summarizes the evidence from the authors' own research, now extending over three decades, into employment, unemployment and welfare benefits across the UK and adds a quantitative overview of the contemporary labor market in Britain's older industrial towns, drawing on official statistics and the authors' most recent research. Job losses, labor market adjustment and the contemporary labor market in older industrial towns have been covered in an earlier report (Beatty and Fothergill, 2018). Here the empirical evidence has been comprehensively up-dated, mostly from 2016 to 2019, and the conclusions revised accordingly.

## Background

Because so many of Britain's industrial job losses happened a generation or more ago and because so many of the redundant workers have themselves reached pension age or died it has become easy to assume that the problems arising from deindustrialization have passed into history. Indeed, as the second decade of the twenty first century drew to a close official figures pointed to unemployment levels in the UK that were lower than at any time since the mid-1970s, when large-scale industrial job losses were only just beginning to get underway. If official unemployment data is the preferred guide, then the problems arising from deindustrialization do indeed appear to have been overcome.

There are however more dimensions to labor market disadvantage than just unemployment, and national figures almost always hide important differences between places. Britain's older industrial towns—the smaller places beyond the big cities that generally have an industrial history extending far back into the nineteenth century—are where so much of British industry was once concentrated. These are the places where the loss of industrial jobs is likely to have been most keenly felt and where we might expect to observe lasting impacts. By contrast, although Britain's cities too nearly all have an industrial past they have always played a wider role in regional and local economies as service centers for their hinterlands, administrative headquarters, transport hubs and as home to major universities.

The recession provoked by the 2008 financial crisis was in many ways a wake-up call, prompting a rediscovery of the divergent labor market trends between North and South (Gardiner et al., 2013) and between city regions (Townsend and Champion, 2014; Centre for Cities, 2015; Swinney and Thomas, 2015; Martin et al., 2016; Pike et al., 2016). In particular, there has been a growing realization that Britain's older industrial towns may not after all be well on the way to recovery. In the 2016 referendum on EU membership, for example, older industrial towns in England and Wales generally voted ‘leave’ by a margin of two-to-one (Jennings, 2017). This has been widely interpreted as a reflection of rising disaffection and disenchantment with the economic impacts of globalization and deindustrialization. The big cities, by contrast, mostly voted ‘remain.’

As the realization of on-going problems has grown, the term ‘left behind places’ has gained widespread use in British political debate and it has been applied in particular to older industrial towns. A sign of shifting priorities has been the UK government's announcement of Town Deals, which make available additional funding for economic and social development (Ministry of Housing, Communities and Local Government, 2019). Of the first 100 towns across England invited to submit bids to the new fund, rather more than half could be described as ‘older industrial.’

Britain is not unique of course in having towns that have lost most or all of their original industrial base. Deindustrialization also characterizes parts of North East France, the former East Germany and the ‘rustbelt’ of the United States, to mention just three other examples. In Britain, however, the original industrialization happened earlier and the efforts to rebuild economies have mostly been in place longer. Reflecting the research on which it is based, the evidence in this paper focusses exclusively on Britain but there may well be pointers to the experience in other developed, post-industrial economies.

## Structure of the Paper

The next section of the paper summarizes the industrial job losses that have taken place in recent decades in Britain, and their distinctive geography. The following section looks at how the labor market adjusted to these job losses, highlighting the extent to which the impacts have been on labor force participation rather than recorded unemployment.

The core of the paper then looks at the contemporary labor market in Britain's older industrial towns, presenting a range of new evidence covering employment, unemployment and economic inactivity, industry mix, job quality, skills, pay and welfare benefits. This is preceded by a short section on a working definition of the towns, necessary in order to deploy a range of official statistics.

The final part of the paper considers the labor market links between older industrial towns and neighboring cities, before concluding with an assessment of the position that Britain's older industrial towns occupy in the economy of the early twenty first century.

## The Destruction of Industrial Britain

Employment in British manufacturing peaked in 1966 when 8.9 million people worked in this sector, accounting for 30% of all employment. By 2019, the UK government's Office for National Statistics put the numbers employed in manufacturing at just 2.7 million, or 7.7% of the employed workforce.

UK manufacturing employment fell especially steeply in the early 1980s during a recession triggered by a high exchange rate and high interest rates. The job losses during these years were documented in particular by Townsend (1983) and Fothergill and Guy (1991) and the process of deindustrialization more generally by Martin and Rowthorn (1986). The recession of the early 1990s added further major job losses (Gudgin, 1995). Thereafter, manufacturing output largely stagnated and manufacturing employment continued to slide even though the UK economy as a whole enjoyed 15 years of sustained economic growth. The recession triggered by the 2008 financial crisis reduced UK manufacturing employment still further and during the subsequent economic recovery manufacturing employment did no more than stabilize at a new lower level.

The coal industry's job losses go back much further. When UK coal production peaked in 1913, 1.1 million miners were employed in over 3,000 mines. For much of the rest of the twentieth century employment in the UK coal industry fell, though there were still 450,000 miners working in the mid-1960s. The more recent job losses began in earnest after the year-long miners' strike of 1984/5, which failed in its attempt to stop pit closures, and the final colliery closed in 2015.

The shift from industrial to service sector employment is not unique to the UK. In all advanced economies it has its roots in differential rates of growth in labor productivity—it is generally easier to replace people by machines in manufacturing than in most service activities—and the shift away from industry has been accentuated by globalization, which has resulted in much routine production moving to China and other emerging economies. In the UK, however, the process of deindustrialization has probably gone further and faster than elsewhere.

**Figure 1:**
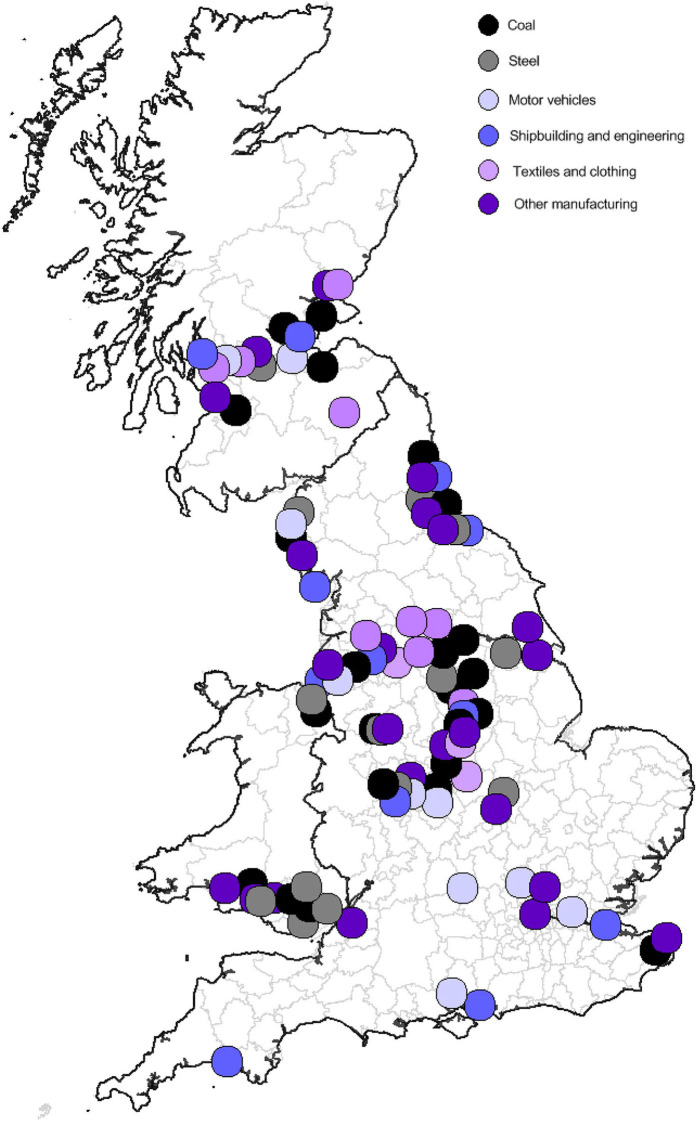
Major industrial job losses across Britain since the early 1980s. Source: Beatty and Fothergill (2017).

The UK's industrial job losses have been concentrated in specific parts of the country. Partly this reflects the distribution of manufacturing, which was always more important in some places than others, but partly it reflects the location of the industries such as coal, steel, shipbuilding, heavy engineering and textiles and clothing that experienced the biggest reduction in employment. Figure 1 illustrates the geography of this job loss. This map flags up the most significant job losses, in places where major industries have been reduced to a fraction of their former size or disappeared entirely. The closure of individual large plants accounts for some of these job losses but more often, in the case of the coal and textile industries for example, the job losses affected several sites across neighboring towns.

Manufacturing employment has fallen in just about all parts of the UK but the concentration of industrial job losses north of a line from the Severn estuary to the Wash is especially noticeable. It is the cities, towns and coalfield areas of the Midlands, North, Scotland and Wales that have been hit hardest—a pattern that will be familiar to anyone with a basic knowledge of Britain's economic geography.

## Labor Market Adjustment

The first and most obvious consequence of the large-scale loss of industrial jobs was a rise in the number claiming unemployment benefits. This hovered around 3 million for several years in the 1980s but fell away after the recession of the early 1990s, eventually to less than 1 million for most of the 2000s.

By the end of the 1990s, however, it was clear that the job shortfalls across Britain were highly uneven and that they were no longer accurately reflected by unemployment data (MacKay, 1999; Webster, 2000; Erdem and Glyn, 2001). The present authors' research on the coalfields shed important new light. In this part of Britain, the mines had mostly closed but claimant unemployment was no higher than when the mines had been working. The evidence on the coalfields showed that the main consequence of job loss was in fact a diversion of working age men into ‘economic inactivity’ and in particular into what the Census called ‘permanent sickness’—in practice a withdrawal from the labor market onto incapacity benefits (Beatty and Fothergill, 1996).

A decade later, a follow-up study (Beatty et al., 2007) identified growing job creation in the former coalfields but confirmed the observation that the principal labor market adjustment in response to the loss of mining jobs was an increase in economic inactivity among working age men. By this stage many of the ex-miners had themselves reached state pension age so it was clear that the continuing high level of economic inactivity among men must be spread more widely across the local workforce. Indeed, it appeared that job loss for one generation was being passed on as higher economic inactivity among the next.

The labor market adjustments in the coalfields were not unique. Across the whole of older industrial Britain, from the mid-1980s through to the early 2000s, the numbers out of the labor market—‘economically inactive’—on incapacity benefits surged to unprecedented highs. This triggered the argument that much of the increase was a form of ‘hidden unemployment’—men and women who in a fully employed economy might have been expected to be in work but whose health problems or disabilities entitled them to incapacity benefits instead of unemployment benefits (Beatty and Fothergill, 2005).

The increase in the numbers claiming incapacity benefits occurred among women as well as men. At first this seemed hard to understand because many of the older industries shedding jobs—coal and steel for example—had a predominantly male workforce. The explanation turned out to be that in older industrial areas and elsewhere the male and female sides of the labor market interact, so the competition for jobs transmits a shortfall in opportunities for men into a difficult labor market for women in the same places. When women with health problems or disabilities are out-of-work they generally then claim incapacity benefits in the same way as their male counterparts (Beatty et al., 2009; Beatty, 2016).

**Figure 2:**
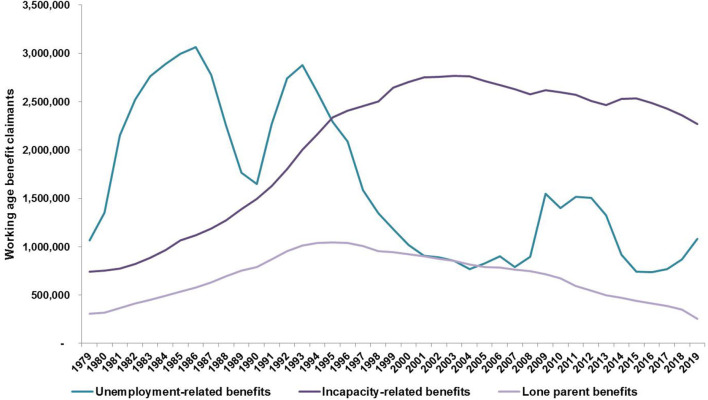
Working age benefit claimants, GB, 1979–2019. Source: Department for Work and Pensions.

Figure 2 shows the numbers claiming the three main out-of-work benefits across Britain as a whole between 1979 and 2019. By the end of this period, and before the recession triggered by the coronavirus crisis, the numbers claiming unemployment-related benefits were well down on the levels of the 1980s and early 1990s, though there was an upturn in the late 2010s resulting from the introduction of the UK's new all-encompassing benefit, Universal Credit, which counts partners and some on small hours or very low pay. The numbers claiming lone parent benefits peaked in the mid-1990s, when the evidence pointed to job loss among men as a driving factor (Rowthorn and Webster, 2008), but have fallen back as eligibility has been restricted just to those with the very youngest children and, more recently, as Universal Credit has been phased in.

The striking feature of Figure 2 is the rise in the numbers out-of-work on incapacity-related benefits, these days Employment and Support Allowance or, as the changeover takes place, Universal Credit on the grounds of ill health or disability. The numbers on these benefits rose from around 750,000 to a plateau of more than 2.5 million in the early 2000s and have subsequently only fallen to around 2.25 million. It is impossible to explain the large increase in health terms alone at a time when general standards of health and physical well-being have slowly been improving.

The highest incapacity claimant rates are predominantly in older industrial Britain. Exposure to industrial injury and disease means that older industrial Britain has long had higher levels of incapacitating ill health but the surge in incapacity claimant numbers only occurred after the industrial jobs began to disappear. Ill health or disability is not always an absolute bar to employment, so what seems to have happened is that where there are plenty of jobs the men and women with health problems or disabilities have been able to hang on in employment or find new work if they are made redundant. However, where the labor market is more difficult, as in much of older industrial Britain, ill health or disability has ruined many people's chances of finding and keeping work.

In effect, the job loss in older industrial Britain has led to higher numbers out-of-work on benefits but not in ways that were perhaps expected. It is the numbers on incapacity benefits, not unemployment benefits, that in the long-run have been impacted most.

Although the rise in incapacity claimant numbers is the defining feature of labor market adjustment in much of older industrial Britain the phenomenon is now past its peak. The national reduction in incapacity numbers since the early 2000s has occurred particularly in older industrial areas, where the claimant rate has typically fallen from above 10% of all adults of working age to a new average of 7–8%, though still well above the 3–4% in the more prosperous parts of southern England. The most recent estimate of ‘hidden unemployment’ among incapacity-related claimants, for 2017, puts the figure at 760,000, well down on an estimated peak of 1.15 million in 2002 (Beatty et al., 2017).

That incapacity numbers in older industrial Britain have fallen back from peak levels owes something to job growth in these places. In the former coalfields, for example, the number of employee jobs increased by 138,000 between 2012 and 2017 (Beatty et al., 2019). The wider growth in UK employment since 2010 has also provided commuting opportunities, particularly in nearby cities. Falling incapacity numbers probably also owe something to revised medical assessments, more restrictive entitlements and new conditionality for some, though the increase in women's state pension age has pushed in the opposite direction, bringing an additional cohort of over-60s into the scope of incapacity-related benefits.

## The Contemporary Labor Market in Britain's Older Industrial Towns

### A Working Definition of the Towns

So what exactly does the labor market now look like in Britain's older industrial towns? The figures we are able to present here are mostly for 2019—at the end of a sustained period of recovery from the 2008 financial crisis but before the coronavirus crisis hit economies across the world. The recession triggered by coronavirus will have worsened labor market conditions just about everywhere but its impact specifically on Britain's older industrial towns will take some while to become clear. It seems unlikely, however, that deep-seated differences between places, and in particular between older industrial towns and more prosperous local economies, will be easily overturned.

Presenting quantitative evidence on the labor market in Britain's older industrial towns requires the use of local area statistics, which in turn requires a working definition of the places to be included. Although these towns are a substantial part of the country there is no official definition of them to assist in the deployment of statistics. In this article we therefore use the term ‘older industrial towns’ to include all Britain's older industrial areas beyond the main regional cities, and we use district and unitary local authorities as the building block because they are the smallest unit for which much of the contemporary labor market data is available. The list of authorities is shown in Table 1. The list is taken from our earlier report on older industrial towns (Beatty and Fothergill, 2018) and has also been deployed in a recent study of labor market adjustment (Beatty and Fothergill, 2020), where a fuller description can be found.

**Table 1:** Districts and unitary local authorities covering Britain's older industrial towns.

**North East**	**Yorkshire and Humber**	**Scotland**
County Durham	Barnsley	Clackmannanshire
Darlington	Bradford	Dundee
Gateshead	Calderdale	East Ayrshire
Hartlepool	Doncaster	East Dunbartonshire
Middlesbrough	Hull	East Lothian
North Tyneside	Kirklees	East Renfrewshire
Redcar and Cleveland	NE Lincolnshire	Falkirk
South Tyneside	North Lincolnshire	Fife
Stockton on Tees	Rotherham	Inverclyde
Sunderland	Wakefield	Midlothian
		North Ayrshire
		North Lanarkshire
**North West**	**East Midlands**	Renfrewshire
Allerdale	Amber Valley	South Lanarkshire
Barrow in Furness	Ashfield	West Dunbartonshire
Blackburn with Darwen	Bassetlaw	West Lothian
Bolton	Bolsover	
Burnley	Chesterfield	
Bury	Corby	**Wales**
Chorley	Erewash	Blaenau Gwent
Copeland	Gedling	Bridgend
Halton	Mansfield	Caerphilly
Hyndburn	Newark and Sherwood	Carmarthenshire
Knowsley	NE Derbyshire	Flintshire
Oldham		Merthyr Tydfil
Pendle		Neath Port Talbot
Preston	**West Midlands**	Newport
Rochdale	Dudley	Rhondda Cynon Taf
Rossendale	Newcastle under Lyme	Swansea
Salford	Sandwell	Torfaen
Sefton	Stoke on Trent	Wrexham
South Ribble	Walsall	
St Helens	Wolverhampton	
Stockport		
Tameside		
Trafford		
Warrington		
Wigan		
Wirral		

*Source: Beatty and Fothergill (2020)*.

The core of the list comprises the former coalfields of the Midlands, North, Scotland and Wales, where so much of the UK's early industrialization took place and where so many of the UK's older industries once flourished. The additions to this core cover the locations of job loss from the UK's main steelworks, shipyards and concentrations of heavy engineering and chemicals. The list also includes the former mill towns of Lancashire and West Yorkshire, where the textile industry has all but disappeared. All the local authorities are in parts of the country where job losses from older industries have long posed a problem, in contrast to southern England where older industries were generally a smaller component of the economy. In 2018 the local authorities on the list had a combined population of 16.8 million, or 26% of the GB total.

Four points are worth noting. First, some of the local authorities actually cover substantial cities—Sunderland, Hull, Bradford, Stoke and Swansea are examples—though none of these are the main city within their region. Second, a number of the local authorities cover numerous quite small towns, especially in former mining areas such as County Durham. Third, some of the local authorities include rural areas just as some of the obvious omissions (Northumberland is an example) include sub-areas that are older industrial in character. Fourth, the sheer extent of job loss over the years means that in some cases ‘industrial’ may no longer be a very good description of the present-day town.

### Labor Market Status

It is useful to begin by looking in Table 2 at the labor market status of working-age (16–64 years old) residents in Britain's older industrial towns. The figures here show that of the more than 10 million working age residents in the towns, 7.5 million were in work in 2019—an employment rate of 73%.

**Table 2:** Labor market status of 16–64 years olds in older industrial towns, 2019.

	**No**.	**%**
In employment	7,510,000	73.1
ILO unemployed	380,000	3.7
Students	540,000	5.3
Looking after family/home	560,000	5.5
Temporary and long-term sick	730,000	7.1
Retired	300,000	2.9
Other	230,000	2.2
All 16–64 year olds	10,270,000	100.0

*Source: Annual Population Survey*.

By comparison, recorded unemployment was modest—just 380,000, equivalent to 3.7% of the working age population. The unemployment figure here comes from the government's Annual Population Survey and uses the International Labour Organisation (ILO) definition of unemployment, which counts anyone who is out-of-work, available to start work within 2 weeks and has looked for work within the last 4 weeks. The ILO unemployment figures do not depend on benefit status and in the years following the financial crisis have been notably higher than UK claimant count. The 3.7% of the workforce unemployed in older industrial towns on the ILO measure is therefore actually the higher of the UK's two official unemployment estimates, which underlines the extent to which recorded unemployment had receded prior to the coronavirus crisis.

The remaining adults of working age fall into a number of categories. In Britain's older industrial towns there were rather more than 500,000 students, many of whom will be still at school or college rather than in higher education. There were more than 500,000 who look after family or home on a full-time basis, and 300,000 who described themselves as ‘retired.’

At 730,000, and accounting for 7.1% of all adults of working age, the single largest group of among the non-employed in the towns were the temporary and long-term sick, of whom all but 50,000 were in the long-term category. The Annual Population Survey tends to under-record the size of this group: in older industrial towns in May 2019, Department for Work and Pensions benefit data put the number of 16–64 year olds out-of-work and claiming incapacity-related benefits at 776,000, or 7.5% of all adults of working age. These figures underline the point that a diversion out of the labor market onto incapacity benefits remains a key feature of the towns. Indeed, with roughly a quarter of the GB population, Britain's older industrial towns are home to more than a third of the country's incapacity-related claimants.

Table 3 draws comparisons between older industrial towns, the main regional cities, London and the GB average. There are similarities and important differences. On the ILO measure of unemployment there is little to differentiate older industrial towns: the rate in 2019 was a little higher than the national average but a little below the level in the main regional cities. In contrast, the incapacity-related claimant rate in older industrial towns (7.5%) was higher than the rate in the main regional cities (7.1%) and well above the GB average (5.7%) and the rate in London (4.4%).

**Table 3:** Labor market status: comparisons, 2019.

	**Older industrial towns** **(%)**	Main regional cities^*^ **(%)**	**London** **(%)**	**GB** **(%)**
ILO unemployment rate	3.7	4.2	3.8	3.3
Incapacity-related claimant rate^**^	7.5	7.1	4.4	5.7
Employment rate	73.1	69.9	74.5	75.6
Students	5.3	9.7	7.0	5.7
Employment rate excluding students	77.2	77.4	80.1	80.1

* *Birmingham, Cardiff, Edinburgh, Glasgow, Leeds, Liverpool, Manchester, Newcastle upon Tyne, Nottingham and Sheffield (all defined as their local authority district)*.

** *May 2019. Includes individuals with limited capacity to work transferred to Universal Credit*.

*All figures are percentages of 16–64 year old residents*.

*Sources: Annual Population Survey, Department for Work and Pensions*.

The employment rate—the share of adults of working age in work—paints a complex picture. On the raw figures, the employment rate in older industrial towns in 2019 does not appear unduly low—a little lower than the GB average or than in London but distinctly higher than in the main regional cities. The raw figures are however misleading. The big distortion is the distribution of students across the country, who are concentrated in London and the main regional cities where so many universities are located. In older industrial towns students accounted for just over 5% of all 16–64 year olds; in the main regional cities they accounted for nearer 10%. This distortion to the figures has always existed but as student numbers have increased it has become more important.

A better measure is therefore the *employment rate excluding students*. This points to older industrial towns as a whole lagging three percentage points behind the national average. It also narrows the gap between older industrial towns and the main regional cities. On this measure the labor market in older industrial towns looks less convincingly healthy.

### Employment Structure

Table 4 shows the sectoral breakdown of the jobs in older industrial towns. In total 6.4 million jobs were located in the towns in 2019. Manufacturing, energy and water—‘industry’—accounted for one-in-seven, or 950,000 jobs in total. These days employment in older industrial towns is dominated by the service sector, particularly by over 2 million jobs in education, health and public administration, which will mostly be in the public sector, and by retail, distribution, hotels and related activities (a further 1.2 million in 2019).

**Table 4:** Industry breakdown of jobs in older industrial towns, 2019.

	**No**.	**%**
Manufacturing, energy and water	950,000	14.7
Construction	460,000	7.1
Retail, distribution, hotels etc.	1,260,000	19.6
Transport and communications	510,000	7.9
Banking, finance and business services	810,000	12.6
Education, health and public admin	2,080,000	32.4
Other services	330,000	5.2
All jobs	6,440,000	100.0

*Source: Annual Population Survey*.

The number of jobs in public services, such as schools and hospitals, is in most places driven by the size of the local population and many jobs in other parts of the service sector, such as retailing, follow local spending power, which is population-related. By contrast, jobs in businesses that serve markets beyond the local area, including most manufacturing but also some parts of the service sector, play a key role in driving the whole local economy because they bring in income to an area which then recirculates and supports other local businesses and jobs. Manufacturing's on-going significance to the economy of older industrial towns is therefore substantially greater than its share of employment.

Table 5 compares employment in older industrial towns with the main regional cities, London and the national average. This bears out the point that jobs in several parts of the service sector—retailing and public services for example—are found in large numbers in all areas because they are tied to population. The differences between older industrial towns and other places are in other sectors.

**Table 5:** Industry breakdown of employment: comparisons, 2019.

	**Older industrial towns** **(%)**	**Main regional cities** **(%)**	**London** **(%)**	**GB** **(%)**
Manufacturing, energy and water	15	8	4	11
Construction	7	7	7	7
Retail, distribution, hotels etc.	20	17	14	18
Transport and communications	8	9	13	9
Banking, finance and business services	13	20	29	18
Education, health and public admin	32	34	26	30
Other services	5	5	7	6
All jobs	100	100	100	100

*Source: Annual Population Survey*.

In particular, despite years of decline which has often led to the disappearance of whole industries, Britain's older industrial towns continue to have a higher proportion of jobs in industry than the economy as a whole, than the main regional cities or than London in particular. These may be ‘older industrial towns’ but they remain to a large extent the heartland of British industry. The converse is that older industrial towns have proportionally fewer jobs in banking, finance and business services than either the main regional cities or London. The drivers of the economy of older industrial towns remain very different from those in London or the main regional cities.

### Job Quality and the Workforce

In older industrial towns the shift from mining and manufacturing to new forms of employment has inevitably resulted in major changes in the experience of work. The qualitative aspects of these changes—job satisfaction, a sense of identity, and control over tasks and workload for example—are very real no doubt, and deserving of study. Here we are limited to the insights that official statistics are able to offer.

Table 6 brings together a number of indicators, again for 2019. These cover the nature of the employment in older industrial towns and two measures of the local workforce—the proportion of working age residents with degree-level qualifications and the proportion born outside the UK.

**Table 6:** Selected labor market indicators: comparisons, 2019.

	**Older industrial towns** **(%)**	**Main regional cities** **(%)**	**London** **(%)**	**GB** **(%)**
Self-employed (% of employed residents)	11	12	18	14
Part-time work (% of jobs in area)	25	22	19	25
White-collar jobs^*^ (% of jobs in area)	39	50	60	47
Degree or equivalent^**^ (% of 16–64 year olds)^***^	31	41	53	39
Born outside UK (% of 16–64 year olds)^***^	10	23	44	19

* *Managerial, professional, associate professional and technical occupations*.

** *NVQ level 4 or higher*.

*** *Data for year to end 2018*.

*Source: Annual Population Survey*.

One of the widespread assumptions about the UK labor market is that as the economy recovered from the recession caused by the 2008 financial crisis the growth in employment was skewed toward part-time and insecure working, including debased forms of self-employment. A common assumption, too, is that these forms of employment became particularly prevalent in weaker local economies, such as much of older industrial Britain, where welfare reforms made it increasingly difficult for many claimants to stay on benefits. The proliferation of ‘self-employed’ delivery workers and taxi drivers, for example, was in the popular view been a defining feature of the last decade.

The first line of Table 6 shows that in fact self-employment in older industrial towns accounts for a below national average share of the workforce and a considerably smaller proportion than in London. This snapshot for 2019 does not however tell the full story because ‘self-employment’ has gradually been increasing. Expressed as a proportion of all employed residents, the increase since 2010 in older industrial towns was only 1 percentage point, on top of a 1 percentage point increase between 2000 and 2010, but the absolute numbers are substantial—an increase of 140,000 between 2010 and 2019. Self-employment accounted for 30% of the increase in residents in employment in older industrial towns between 2010 and 2019.

The increase in self-employment is probably unwelcome. As the UK government itself has documented (Department for Business, Innovation and Skills, 2016), the self-employed as a group have seen falling incomes since the post-financial crisis recession, which mostly reflects their changing composition as a group. The modern self-employed worker is less likely to be a prosperous entrepreneur or freelance worker than a quasi-employee with diminished employment rights.

The second line of Table 6 deals with part-time employment. This points to a share of jobs in older industrial towns that is only a little higher than in the main regional cities and in line with the GB average, and in older industrial towns the share fell by 1 percentage point between 2010 and 2019. Once again, the raw figures do not tell the whole story. According to the government's Annual Population Survey, 2.5 million people across the UK as a whole were ‘underemployed’ in 2019 in that they wanted to work more hours, were able to start to do so within 2 weeks and were already working less than full-time. This was down on the peak level of around 3.1 million in the wake of recession but still higher than the pre-recession figure of just below 2 million. Across the UK, around one-in-ten part-time workers say they could not find a full-time job.

Additionally, there has been an increase in the number of employees on zero hours contracts (Office for National Statistics, 2018). A government survey of businesses puts the figure for 2017 at 1.8 million contracts that did not guarantee a minimum number of hours. The Annual Population Survey puts the national figure for 2019 at 900,000, or 2.7% of all people in employment. Since 2010 the numbers have risen sharply from around 200,000 but the Office for National Statistics takes the view that part of the observed increase appears to be due to increased recognition and awareness of this form of employment. No local figures are available. According to the Office for National Statistics, the people on zero hours contracts are more likely to be young, part-time, women or in full-time education when compared with other people in employment, and only around a quarter say they would like more hours, mostly in their current job.

Across the UK as a whole, the Annual Population Survey points to 3% of workers with second jobs, and 4% in temporary employment. Of those in temporary employment, a quarter say this is because they could not find a permanent job, a proportion that has fallen from around 40% in the immediate wake of the financial crisis. Again, no local figures are available but the small national percentages with second jobs or in temporary employment suggest that both are likely to be relatively marginal features of the labor market in older industrial towns, though that does not rule out the possibility of increases since the pre-financial crisis years.

But if self-employment and part-time working (and possibly zero hours contracts, second jobs and temporary working) do not sharply differentiate older industrial areas from other places the remaining indicators in Table 6 most certainly do. The share of white-collar jobs is far below the level in the cities, the proportion of the workforce educated to degree level is far lower and so is the proportion of the workforce born outside the UK.

There are issues here of cause-and-effect. One interpretation could be that it is the location of white-collar jobs that follows the location of highly educated workers. There will always be cases that fit this model. A more likely model is that the composition of the workforce in older industrial towns reflects the nature of the job opportunities and that there is a migration of highly-educated workers out of the towns to the cities where they are more likely to find appropriate employment. Some of this will occur as school-leavers from the towns move to university, mostly in the cities, and then never return. At the extreme, London's exceptionally high proportion of graduates clearly reflects the availability of higher-level jobs that attract graduates from elsewhere in Britain and from the rest of the world. The industrial and service jobs that form such a large component of the economy in older industrial towns do not have the same magnetic appeal.

Likewise, the low proportion of the workforce born outside the UK at least in part reflects the long-term weakness of the economy in older industrial towns. Migrants are attracted to the places where jobs are more readily available. It should be no surprise, therefore, that international migrants are fewer in number in towns where the economic base has been eroded. There are exceptions of course—Bradford and the Lancashire mill towns are examples, where there continues to be in-migration to established Asian communities. As a general rule, however, it is the strength of the local economy to which we should look for the prime explanation.

### Pay and Welfare Benefits

Table 7 compares the median weekly earnings of employees in older industrial towns with the equivalent figures for the main regional cities, London and the GB average. The first part of the table shows the figures for the jobs located in the area and demonstrates that in older industrial towns these jobs pay less than the national average, less than in the main regional cities and substantially less than in London. The median earnings of jobs in London are almost £200 a week (or 45%) higher than in older industrial towns.

**Table 7:** Median earnings: comparisons, 2019.

	**Older industrial**	**Main regional**	**London**	**GB**
	**towns**	**cities**		
**Jobs in area**				
Gross weekly pay	£ 438	£ 481	£ 634	£ 479
GB = 100	91	100	132	100
**Residents**				
Gross weekly pay	£ 449	£ 446	£ 589	£ 479
GB = 100	94	93	123	100

*Source: Annual Survey of Hours and Earnings*.

The second part of the table shows the earnings of residents. On this measure, older industrial towns are still below the national average and well below the level in London, though in both cases by a smaller margin, but residents' earnings are much the same as in the main regional cities. The differences between the two halves of the table reflect the influence of commuting: some of the residents of older industrial towns fill higher-paid jobs in the cities.

One of the consequences of low pay in older industrial towns is that there is a substantial financial burden on the Exchequer. This occurs because the UK tax and benefit system operates to prop up household incomes not just for those out-of-work but also for those in low-paid employment. To illustrate this point, Table 8 looks at in-work households in receipt of Tax Credits. These figures are for the 2015/16 financial year—the last before Tax Credits began to be subsumed into Universal Credit and as a result difficult to disentangle as a separate category—but it is unlikely that the geography and to some extent the magnitude of the payments will have changed radically.

**Table 8:** In-work households in receipt of Tax Credits, 2015/16.

	**Older industrial towns**	**Main regional cities**	**London**	**GB**
No. of households in receipt	903,000	303,000	386,000	2,932,000
GB = 100^*^	120	108	89	100
Average annualized value (£)	6,479	6,922	7,693	6,726
Total expenditure (£bn)	6.0	2.2	3.0	19.7
Expenditure per 16–64 year old (£)	576	559	498	490

* *No. of households in receipt relative to working age population*.

*Source: Her Majesty's Revenue and Customs*.

The absolute numbers for older industrial towns are large. In total, just over 900,000 in-work households in the towns received an annualized average of almost £6,500 in Tax Credits, at a cost to the Exchequer of £6bn a year. What is also clear is that the cost to the Exchequer of Tax Credits is greater in older industrial towns than in the cities. This is not because the size of the average claim is higher—in fact it is lower in older industrial towns than in the cities—but because low wages bring larger numbers of households into the scope of Tax Credits. Averaged across the whole of the working age population (the final line of Table 8), the expenditure on Tax Credits is higher in older industrial towns than in the main regional cities or London, and higher than the GB average.

## Relationship to the Cities

In recent years the dominant view within economic geography (Jacobs, 1986; Krugman, 1991; Centre for Cities, 2015) and in policy making (HM Government, 2011; Core Cities, 2013) has been that cities are the motor of regional and local growth. By implication, surrounding industrial towns are increasingly expected to function as their satellites, providing a source of labor and an overspill location for businesses. This is very different from the way in which Britain's industrial towns first developed, when they were nearly all locations of business growth in their own right.

What is undeniable is that approaching three-quarters of the population of Britain's older industrial towns listed earlier in Table 1 live in the immediate hinterland of the main regional cities. Only the remaining quarter live in towns located further afield—places such as Barrow in Cumbria or Grimsby on the south bank of the Humber, or smaller cities such as Hull, Swansea, Dundee, and Stoke on Trent that stand at some distance from the main regional cities.

The evidence on job growth in the final years of the twentieth century and the early years of the 21st did not support the view that employment in the UK's main regional cities was growing consistently faster than elsewhere (Champion and Townsend, 2011, 2013; Martin et al., 2014). More recent trends, however, show that job growth in the cities is now substantially faster than in Britain's older industrial towns. As Table 9 shows, this is especially marked when the growth is expressed in relation to the resident working age population. On this measure, between 2010 and 2019 job growth in the main regional cities was five times faster than in older industrial towns, and nearly six times faster in London.

**Table 9:** Increase in employment in area, 2010–2019.

	**No**.	**As % of working age pop**.
London	900,000	16.3
Main regional cities	500,000	14.2
*Great Britain*	*2,880,000*	*7*.*4*
Older industrial towns	290,000	2.8

*Source: Annual Population Survey*.

In a small country such as the UK, with high car ownership and a network of public transport, the labor markets of cities and surrounding towns are inevitably strongly intertwined, as Swinney et al. (2018) have documented. This can be an asset for some older industrial towns if they are close to a city with a strong economy but a problem for others if they are further away or if their neighboring city is less prosperous. Additionally, Pike et al. (2016) identified ‘overshadowed cities’ (most of which are actually large towns)—that is, cities with a larger neighbor that hosts higher-level functions and provides development opportunities—as one of the categories of UK places facing relative decline.

The labor market links between older industrial towns and neighboring areas are certainly strong. Annual Population Survey data for 2019 puts the total net commuting out of older industrial towns at 1.06 million, equivalent to 14% of all residents in employment. Not all the commuting will have been into the main regional cities but this figure is a *net flow*—the daily flow outwards will be significantly larger, offset in part by in-commuting. Looking at commuting flows from the other direction, in 2019 the 10 main regional cities had a net in-flow of 990,000 commuters, equivalent to 27% of all the jobs located there.

Net out-commuting from Britain's older industrial towns rose by 200,000 between 2010 and 2019, and net in-commuting to the main regional cities rose by 100,000 over the same period. Around Manchester, Edinburgh and Cardiff there is clear evidence of rising in-flows of commuters from older industrial towns in surrounding areas (Beatty and Fothergill, 2020) but in these cities the growth in employment has been especially strong and the corresponding growth in their older industrial hinterlands (e.g., in Cardiff's case the Welsh Valleys) has been poor. Elsewhere the gap in job growth has been more modest and the changes in commuting patterns more complex.

London's particularly rapid growth—an additional 900,000 jobs between 2010 and 2019—has however had little impact on older industrial towns. Because of the distances involved it was always unrealistic to expect that London's growth would attract daily commuters from beyond the south of England, though there are undoubtedly Monday-to-Friday flows from longer distance into the capital. London's rapid job growth might however have been expected to attract a net inflow of migrants from older industrial towns in the rest of the country. In practice this has not happened because the London economy has tapped other sources of additional labor: a large net inflow of international migrants, a surge in commuting from the rest of southern England, rising labor force participation among London residents and a natural increase in the size of the local workforce that reflects a population skewed toward younger groups. The balance of internal migration (i.e., the flow of UK residents) has actually been strongly out of London (Beatty and Fothergill, 2020).

London it seems is no longer acting as a powerful magnet for migrants from the older industrial towns of the Midlands, North, Scotland and Wales, in effect detaching its growth from the labor market north of a line from the Severn to the Wash. This observation from the post-financial crisis years confirms a trend first identified in the previous decade: the net flow of internal migrants into London and the South of England has essentially come to a halt (Rowthorn, 2010).

## Assessment

Let us now draw some overall conclusions. The first is that set against the backdrop of years of massive industrial job loss, the labor market in Britain's older industrial towns is not as distressed as might have been feared. In some towns, it is worth remembering, virtually the whole of the original economic base has disappeared and this might have been expected to have resulted in permanent mass unemployment or large-scale depopulation. In fact, taking Britain's older industrial towns as a whole, prior to the coronavirus crisis the unemployment rate was only a little higher than the national average.

Britain's older industrial towns are not, it seems, locked into a spiral of decline. It might have been expected that the loss of most of their former industrial base, which in historical terms happened quite recently, might have triggered a longer-term knock-on loss of jobs, in the local service sector for example as a result of reduced local spending-power. If these negative consequences are still happening in the towns they have so far been offset by other more positive developments, including no doubt the impact of substantial public sector efforts to rebuild their local economies. On balance, the number of jobs in Britain's older industrial towns was growing prior to the coronavirus crisis, though more slowly than elsewhere.

The second conclusion is that even if conditions in Britain's older industrial towns are not as bad as they might have been, the labor market in the towns still remains difficult. This is evident not only in below-par employment rates but also in the very large numbers on incapacity benefits, in low pay, the predominance of manual jobs and a high dependence on in-work welfare benefits. In many respects this is ‘the long shadow of job loss.’

Until very recently, perhaps, the nature of the problem has been different from what it was 20 or 30 years ago. Even allowing for distortions to the official figures, unemployment in the towns was down on peak levels and down on the immediate post-financial crisis years. For many of the workless the problem is therefore likely to have been not that they could not find any job at all, which was probably the case in the era of 3 million claimant unemployed, but rather that they had difficulty finding suitable work with acceptable pay and conditions. In older industrial towns there have simply not been enough of these ‘good’ jobs to satisfy everyone, not least because the destruction of so much industry over so many years has removed the layer of jobs that once filled this important gap in the labor market. The new recession, triggered by coronavirus, looks likely to bring back older difficulties, at least for some while.

The third conclusion is that to describe most of these towns as ‘post-industrial’ is inaccurate. Certainly, they have lost most if not all the industry that underpinned their original growth but the figures here show that manufacturing, energy and water together still account for 15% of local employment—nearly four times the proportion in London and twice the proportion in the main regional cities. Add in the now numerous jobs in warehousing—just another ‘industry’ to many residents, no doubt, and a major element of the economy in several former coalfields—and the proportion would be significantly higher.

The point here is that Britain's older industrial towns remain the heartland of British manufacturing. Unlike the big regional cities, and London in particular, which have shed most of their former industrial jobs and become centers of banking, business services and higher education, many of Britain's older industrial towns remain to an important extent ‘industrial’ with economies that still depend on the local and national performance of this sector.

The fourth conclusion is that Britain's older industrial towns need to be understood in their wider geographical context. They do not exist in isolation from the places around them, in particular from neighboring cities. They are increasingly becoming dormitories for men and women who work elsewhere, partly no doubt because the on-going slack in the local labor market encourages commuting to neighboring areas and further afield.

The scale of commuting should be kept in perspective: the net outflow from the towns is presently just over a million whereas nearly seven and a half million residents of the towns are in work. Moreover, it is not always the big regional cities that are the destination and there is no evidence at all that London's spectacular recent growth has been of any direct benefit to the labor market in Britain's older industrial towns. Nevertheless, commuting on this scale represents a significant redefinition of the role of the towns within wider urban networks.

## Data Availability Statement

The datasets generated for this study are available on request to the corresponding author.

## Author Contributions

All authors listed have made a substantial, direct and intellectual contribution to the work, and approved it for publication.

## Conflict of Interest

The authors declare that the research was conducted in the absence of any commercial or financial relationships that could be construed as a potential conflict of interest.

## References

[B1] Beatty C. (2016). Two become one: the integration of the male and female labour markets in the English and Welsh coalfields. Reg. Stud. 50, 823–834. 10.1080/00343404.2014.943713

[B2] Beatty C. Fothergill S. (1996). Labour market adjustment in areas of chronic industrial decline: the case of the UK coalfields. Reg. Stud. 30, 637–650. 10.1080/00343409612331349928

[B3] Beatty C. Fothergill S. (2005). The diversion from “unemployment” to “sickness” across British regions and districts. Reg. Stud. 39, 837–854. 10.1080/00343400500289804

[B4] Beatty C. Fothergill S. (2017). The impact on welfare and public finances of job loss in industrial Britain. Reg. Stud. Reg. Sci. 4, 161–180. 10.1080/21681376.2017.1346481

[B5] Beatty C. Fothergill S. (2018). The Contemporary Labour Market in Britain's Older Industrial Towns. Sheffield: CRESR, Sheffield Hallam University.

[B6] Beatty C. Fothergill S. (2020). Recovery or stagnation: Britain's older industrial towns since the recession. Reg. Stud. 25, 1–12. 10.1080/00343404.2019.1699651

[B7] Beatty C. Fothergill S. Gore T. (2017). The Real Level of Unemployment 2017. Sheffield: CRESR, Sheffield Hallam University.

[B8] Beatty C. Fothergill S. Gore T. (2019). The State of the Coalfields 2019: Economic and Social Conditions in the Former Coalfields of England, Scotland and Wales. Sheffield: CRESR, Sheffield Hallam University.

[B9] Beatty C. Fothergill S. Houston D. Powell R. Sissons P. (2009). Women on Incapacity Benefits. Sheffield: CRESR, Sheffield Hallam University.

[B10] Beatty C. Fothergill S. Powell R. (2007). Twenty years on: has the economy of the UK coalfields recovered? Environ. Plan. A 39, 1654–1675. 10.1068/a38216

[B11] Centre for Cities (2015). Cities Outlook 2015. London: Centre for Cities.

[B12] Champion T. Townsend A. (2011). The fluctuating record of economic regeneration in England's second-order city regions 1984–2007. Urban Stud. 48, 1539–1562. 10.1177/0042098010375320

[B13] Champion T. Townsend A. (2013). Great Britain's second-order city regions in recession. Environ. Plan. A, 45, 362–382. 10.1068/a45100

[B14] Core Cities (2013). Competitive Cities, Prosperous People: A Core Cities Prospectus for Growth. Manchester: Core Cities.

[B15] Department for Business, Innovation and Skills. (2016). The Income of the Self-Employed. London: BIS.

[B16] Erdem E. Glyn A. (2001). Jobs deficits in UK regions. Oxf. Bull. Econ.Stat. 63, 737–752. 10.1111/1468-0084.63.spe1.7

[B17] Fothergill S. Guy N. (1991). Retreat From the Regions: Corporate Change and the Closure of Factories. London: Jessica Kingsley/Regional Studies Association.

[B18] Gardiner B. Martin R. Sunley P. Tyler P. (2013). Spatially imbalanced growth in the British economy. J. Econ. Geogr. 13, 889–928. 10.1093/jeg/lbt003

[B19] Gudgin G. (1995). Regional problems and policy in the UK. Oxf. Rev. Econ. Policy 11, 18–93.

[B20] HM Government (2011). Unlocking Growth in Cities. London: HM Government.

[B21] Jacobs J. (1986). Cities and the Wealth of Nations. Harmondsworth: Penguin.

[B22] Jennings W. (2017). Cities, Towns and the General Election of 2017. Part 1 of Cities and Towns: The 2017 General Election and The Social Divisions of Place. London: New Economics Foundation.

[B23] Krugman P. (1991). Geography and Trade. Cambridge, MA: MIT Press.

[B24] MacKay R. (1999). Work and nonwork: a more difficult labour market. Environ. Plan. A 31, 1919–1934. 10.1068/a311919

[B25] Martin R. Gardiner B. Tyler P. (2014). The Evolving Economic Performance of UK Cities: City Growth Patterns 1981–2011. London: Foresight, Government Office for Science.

[B26] Martin R. Rowthorn R. (1986). The Geography of De-industrialisation. London: Macmillan.

[B27] Martin R. Sunley P. Tyler P. Gardiner B. (2016). Divergent cities in post-industrial Britain. Camb. J. Reg. Econ. Soc. 9, 269–299. 10.1093/cjres/rsw005

[B28] Ministry of Housing, Communities and Local Government. (2019). Town Deals: Prospectus. London: MHCLG.

[B29] Office for National Statistics (2018). Contracts That Do Not Guarantee a Minimum Number of Hours: April 2018. London: ONS.

[B30] Pike A. MacKinnon D. Coombes M. Champion T. Bradley D. Cumbers A.. (2016). Uneven Growth: Tackling City Decline. York: Joseph Rowntree Foundation.

[B31] Rowthorn R. (2010). Combined and uneven development: reflections on the North–South divide. Spat. Econ. Anal. 5, 363–388. 10.1080/17421772.2010.516445

[B32] Rowthorn R. Webster D. (2008). Male worklessness and the rise of lone parenthood in Great Britain. Camb. J. Reg. Econ. Soc. 1, 69–88. 10.1093/cjres/rsm004

[B33] Swinney P. McDonald R. Ramuni L. (2018). Talk of the Town: The Economic Links Between Cities and Towns. London: Centre for Cities.

[B34] Swinney P. Thomas E. (2015). A Century of Cities: Urban Economic Change Since 1911. London: Centre for Cities.

[B35] Townsend A. (1983). The Impact of Recession on Industry, Employment and the Regions, 1976–1981. Beckenham: Croom Helm.

[B36] Townsend A. Champion T. (2014). The impact of recession on city regions: the British experience, 2008–2013. Local Econ. 29, 38–51. 10.1177/0269094213518885

[B37] Webster D. (2000). The geographical concentration of labour market disadvantage. Oxf. Rev. Econ. Pol. 16, 14–128. 10.1093/oxrep/16.1.114

